# Cardiac rhabdomyoma: an uncommon culprit in sudden infant death

**DOI:** 10.1007/s12024-023-00737-9

**Published:** 2024-07-04

**Authors:** Kori L. Mecklenburg, Erik K. Mitchell, Joseph A. Prahlow

**Affiliations:** 1 Western Michigan University Homer Stryker M.D. School of Medicine, 1000 Oakland Drive, Kalamazoo, MI 49008-8000 USA; 2 Artivion, Kennesaw, GA USA; 3 Department of Pathology, St. Louis University School of Medicine, St. Louis, MO USA; 4 Office of the Medical Examiner - City of St. Louis, St. Louis, MO USA; 5 Department of Pathology and Laboratory Medicine, Cedars Sinai Medical Center, Los Angeles, USA

**Keywords:** Infant, Death, Cardiac tumors, Rhabdomyoma, Forensic pathology

## Abstract

Sudden death in infants remains a common and poorly understood cause of childhood mortality in the USA. Pediatric cardiac tumors, although rare, may underlie some cases of unexplained sudden infant death. Autopsy is a crucial diagnostic step in these cases, as both gross and microscopic examination of the heart may uncover occult cardiac tumors. Rhabdomyomas are the most common cardiac tumors in childhood and may result in arrythmia and sudden death. We present a case of sudden death in a healthy 5-month-old infant which initially appeared “SIDS-like” until thorough histological analysis revealed an underlying cardiac rhabdomyoma. The case is of particular importance in that the gross examination of the heart was considered completely normal, and the tumor only involved certain portions of the heart microscopically. Had a single random section of myocardium been the only heart section examined microscopically, the diagnosis might have been missed. This case emphasizes the importance of thorough microscopic examination in infant cases, especially in cases where the heart appears grossly normal.

## Introduction

Cardiac tumors have a reported frequency of 0.02% and 0.04% within the pediatric population and constitute a rare cause of sudden infant death [1, 2]. Rhabdomyomas represent the most common primary cardiac tumors in children and possess a strong link with tuberous sclerosis [3]. Though considered benign, rhabdomyomas may present with cardiomegaly, congestive heart failure, or sudden perinatal death depending on size and local invasion [4]. This neoplastic striated muscle tissue can contribute to cardiac arrythmia and ultimately death [4]. We report a case of sudden death in an infant discovered at autopsy to have a cardiac rhabdomyoma. This case illustrates the importance of analyzing multiple areas of grossly normal-appearing heart in cases of sudden infant death, as this diagnosis may have been missed had only one myocardial section been analyzed.

## Case information

A 5-month-old female infant was found unresponsive in bed at her caregiver’s home. All resuscitation efforts failed and the infant was pronounced dead. The decedent was last known alive 2 h prior to discovery when she was placed supine and swaddled on the right side of an adult bed. She was alone in bed without co-sleeping. There was no bedding around her. The decedent was placed down for a nap by her babysitter, who was reportedly experienced in childcare.

The decedent’s mother had a history of pre-eclampsia with the decedent’s older sibling and thus took aspirin and an antihypertensive during pregnancy. The decedent was delivered via scheduled cesarean section at 36 weeks with no complications. Following delivery, the child remained at the neonatal intensive care unit for 10 days due to an unknown/undiagnosed “breathing issue.” She received appropriate postnatal care and attended scheduled well-baby visits. Before death, she was believed to be a healthy infant with no known medical issues.

Prior to medicolegal autopsy, and with family permission, the medical examiner approved cardiac valve recovery for transplantation, and also requested a cardiac pathology report, recuts of all histology slides, and return of formalin-fixed tissues to the medical examiner’s office. The infant’s heart weighed 32 g before valve procurement, which is within normal limits for age. No valvular structural abnormalities were noted.

**Fig. 1:**
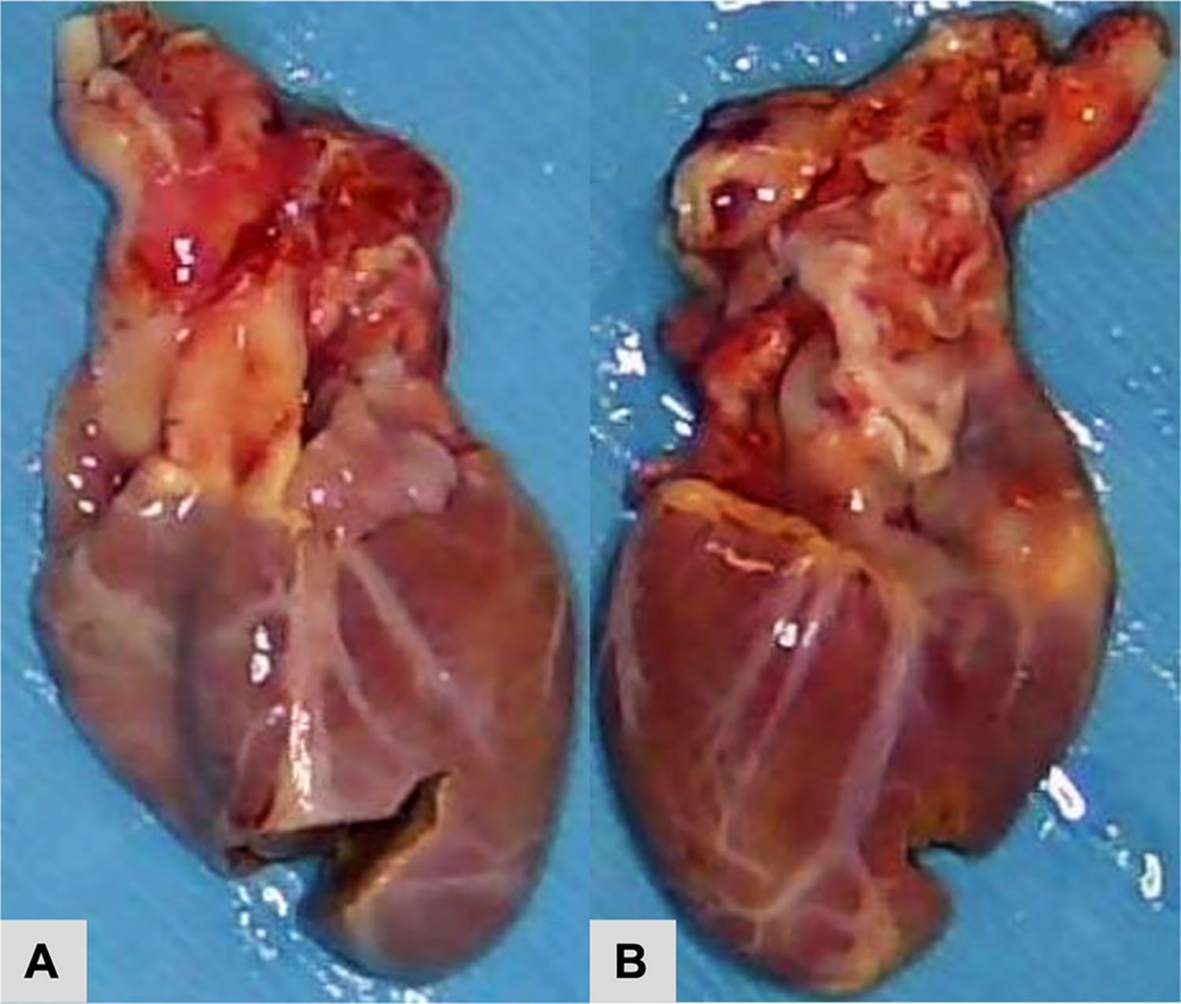
Heart (32 g), unfixed **A** anterior view, **B** posterior view

**Fig. 2:**
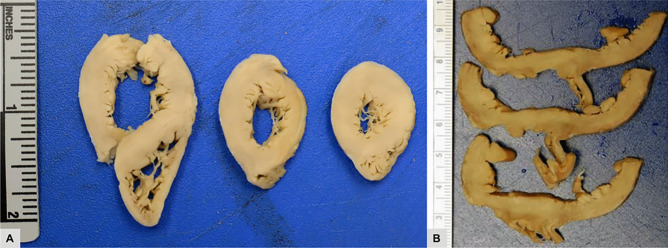
**A** and **B** Ventricular cross sections of formalin-fixed heart, showing absence of abnormality (left ventricle: 0.4 cm, interventricular septum 0.6 cm, right ventricle 0.15 cm)

**Fig. 3:**
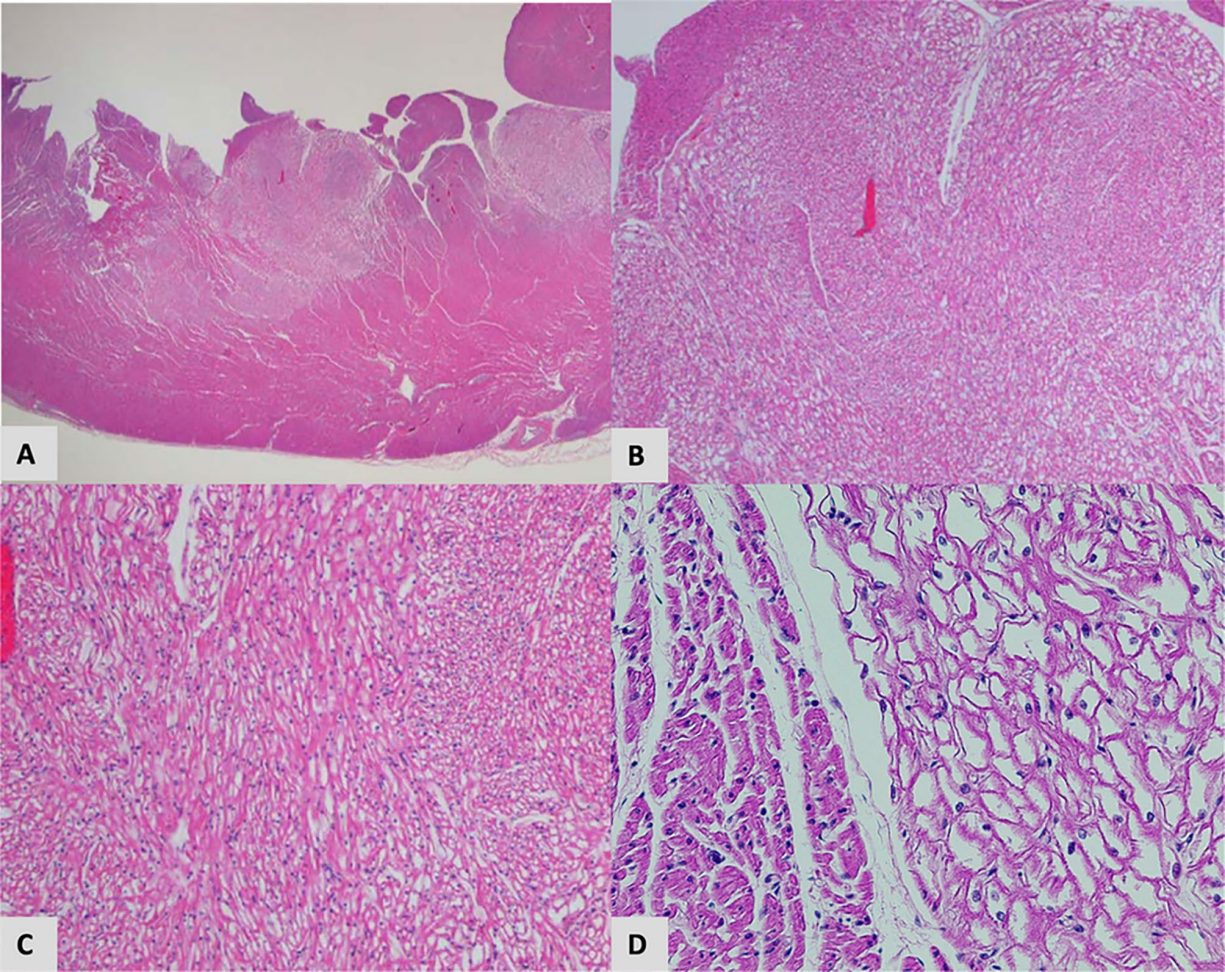
Left ventricle H&E **A** 10× magnification, **B** 40× magnification, **C** 100× magnification, **D** normal myocardium next to tumor (right) at 200× magnification

**Fig. 4:**
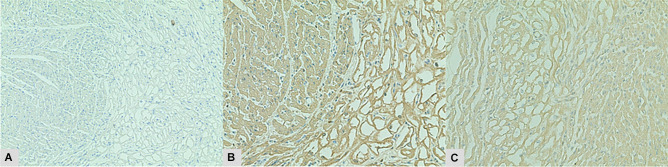
Immunoperoxidase staining: **A** negative S100 staining in normal myocardium and tumor (right) at 100 × magnification, **B** positive myoglobin staining of tumor (right) and normal tissue (200×), **C** positive myogenin immunoperoxidase staining in tumor (left) and normal tissue (200×)

After valve recovery was performed, a full post-mortem examination was conducted at the office of the medical examiner. At autopsy, the infant appeared well-nourished and appropriately developed. Gross examination of the heart revealed no visible pathology (Figs. 1 and 2). Histologic examination revealed multifocal areas of the left ventricular wall with focal left ventricular trabeculae and immediately subendocardial myocardium containing myocytes dilated with uniformly clear cytoplasm (Fig. 3). The neoplastic cells were well-demarcated with clear outlines and nuclei were either central or peripheral within the cell. No pathognomonic spider cells were seen within the samples examined, but cellular findings were ultimately considered consistent with a diagnosis of benign rhabdomyoma. Subsequent immunoperoxidase staining revealed positive staining with myoglobin and myogenin, and negative staining with S100 (Fig. 4). Other sections of the left ventricle, interventricular septum, and right ventricle demonstrated no inflammation, necrosis, fibrosis, hemorrhage, or myocyte disarray. Other organ systems appeared grossly normal, with no significant histopathologic findings. Gross and histologic examination of the brain revealed no abnormalities. There were no other stigmata of tuberous sclerosis identified. Laboratory testing, including newborn genetic screen and toxicology testing, was unrevealing.

The cause of death was certified as left ventricular rhabdomyoma. The manner of death was natural.

## Discussion

In 2020, the infant mortality rate was 541.9 infants per 100,000 live births [5]. Congenital malformations were responsible for the highest number of these infant deaths, followed by low birth weight, followed by “sudden infant death syndrome” [5]. Sudden infant death syndrome (SIDS)—now more commonly referred to by the forensic pathology community as “sudden unexplained death in infancy”—is a broad diagnosis describing unexplained death in an infant less than 12 months old. In 2020, SIDS was responsible for approximately 38 deaths per 100,000 live births [5]. Despite associations with unsafe sleep, the mechanism of SIDS remains poorly understood. Cardiac tumors constitute a rare cause of sudden infant death which may initially resemble SIDS at autopsy [1, 2].

Rhabdomyomas represent the most common primary cardiac tumors in the pediatric population. These benign neoplasms disrupt the cardiac architecture, predisposing to cardiac arrythmia and sudden death [4, 6]. The first case of cardiac rhabdomyoma presenting as sudden infant death syndrome was documented by Rigle in 1989 [7]. Sudden death due to clinically silent rhabdomyoma has also been documented in a handful of cases where the child died during physical activity [4, 7, 8]. It is thought that rhabdomyomas lead to sudden death from arrythmia due to their proximity to the cardiac conduction system [9, 10]. Some large cardiac rhabdomyomas may also cause mechanical obstruction of blood flow, and surgical resection may improve prognosis [9, 11]. In the present case, where the rhabdomyoma was multifocal and non-obstructive, death likely occurred due to fatal arrythmia.

Cardiac rhabdomyomas are defined by a constellation of gross and microscopic features which may be evident during autopsy. Rhabdomyomas present grossly as either single or multiple cardiac masses, preferentially arising in the ventricles [6, 12]. However, these tumors may also appear in the atria, cavoatrial junction, or epicardial surface [13]. Smaller rhabdomyomas may not be visible grossly and instead appear only during histological studies, as in the presented case. Microscopically, rhabdomyomas are classically characterized by “spider cells” consisting of swollen myocytes with clear cytoplasm and abnormal vacuolization. Immunoperoxidase staining can also help identify rhabdomyoma histology, with neoplastic muscle tissue staining positively for myoglobin and myogenin, and negatively for S100 [14–16]. Both rhabdomyomas and their malignant counterparts stain positively for myoglobin and demonstrate a cytoplasmic staining pattern [15]. S100 positivity occurs in certain sarcomas and myoepithelial tumors, and rarely occurs in rhabdomyomas [15]. If positive in rhabdomyomas, S100 demonstrates a focal staining pattern [15]. Myogenin stains positively in fetal striated muscle and would be present in both the rhabdomyoma and surrounding normal cardiac tissue [17]. Interpreted alongside cellular morphology, these immunoperoxidase staining patterns strongly support a diagnosis of rhabdomyoma.

Cardiac rhabdomyomas must be differentiated from other cardiac disorders, including other neoplastic disorders, as well as histiocytoid cardiomyopathy. Histiocytoid cardiomyopathy (HC) is a rare cardiac disorder found in children under 2 years old, with a 4:1 female predominance [18–20]. Clinical features of HC include dysrhythmia, heart failure, and sudden death [18, 20]. Most cases of histiocytoid cardiomyopathy are diagnosed at autopsy, and gross examination of the heart reveals yellow multifocal thickening of the endocardium [20, 21]. Abnormal histiocytoid cells can be visualized microscopically, containing abundant granular, foamy cytoplasm and dark centrally-located nuclei [18, 20]. Electron microscopy confirms that these histiocytoid cells are abnormal myocytes, with prominent Z-bands, large numbers of mitochondria, and very few and distorted myofibrils [20, 21]. Together, these gross and microscopic features can help pathologists distinguish between cardiac rhabdomyoma and histiocytoid cardiomyopathy at autopsy. It should also be noted that, even if routine gross and microscopic examination of the heart in sudden infant deaths appear normal, an explanation for death may be evident by examining the cardiac conduction system microscopically [21]. Though rare, infiltrative vascular disease of conduction system vessels has been documented and may present as sudden infant death [21].

Ultimately, a thorough forensic autopsy including tissue analysis of grossly normal-appearing heart tissue is warranted to uncover pathologic processes which may explain a sudden infant death. Finding rhabdomyomas at autopsy may be the only presenting characteristic of tuberous sclerosis, a genetic condition whose discovery may warrant genetic testing of living siblings. The present case illustrates the importance of histological studies to complement gross findings of a forensic autopsy. It also confirms the importance of cardiac examination in cases approved for valve recovery. The case is of particular importance in that the gross examination of the heart was considered completely normal, and the tumor only involved certain portions of the heart microscopically. Had a single random section of myocardium been the only heart section examined microscopically, the diagnosis might have been missed. As such, it is advisable for forensic pathologists to submit multiple myocardial sections for histologic examination in all sudden unexplained infant deaths, even when the heart is grossly normal. At minimum, a section of the left ventricular free wall, interventricular septum, and right ventricle should be examined in these cases.

## Key points

1. Rhabdomyomas are the most common pediatric cardiac tumors and may underlie some causes of sudden infant death.
2. We present the case of a 5-month-old infant whose sudden death initially appeared “SIDS-like” until histological analysis of multiple myocardial sections revealed a cardiac rhabdomyoma.
3. This case is of particular importance because if a single random section of myocardium had been analyzed, the diagnosis may have been missed.
4. Important differential diagnoses for cardiac rhabdomyoma include histiocytoid cardiomyopathy, cardiac conduction system abnormalities, and other heart neoplasms.
5. A thorough forensic autopsy including tissue analysis of grossly normal-appearing heart is warranted to uncover such rare causes of sudden infant death.
